# Supplementary material for: On-chip phonon-magnon reservoir for neuromorphic computing
Source: Nat Commun. 2023 Dec 14;14:8296. doi: 10.1038/s41467-023-43891-y (PMC10721880; doi:10.1038/s41467-023-43891-y)
Supplement: Supplementary file 1 — Supplementary Information [file 41467_2023_43891_MOESM1_ESM.pdf]

SUPPLEMENTARY INFORMATION

On-chip phonon-magnon reservoir for neuromorphic computing

Dmytro D. Yaremkevich[1], Alexey V. Scherbakov[1], Luke De Clerk[2,6], Serhii M. Kukhtaruk[3], Achim Nadzeyka[4], Richard Campion[5], Andrew W. Rushforth[5], Sergey Savel'ev[2], Alexander G. Balanov[2] and Manfred Bayer[1]

[1]Experimentelle Physik 2, Technische Universität Dortmund, D-44227 Dortmund, Germany.

[2]Department of Physics, Loughborough University, Loughborough LE11 3TU, United Kingdom.

[3]Department of Theoretical Physics, V. E. Lashkaryov Institute of Semiconductor Physics, 03028 Kyiv, Ukraine.

[4]Raith GmbH, 44263 Dortmund, Germany

[5]School of Physics and Astronomy, University of Nottingham, Nottingham NG7 2RD, United Kingdom.

[6]Machine Learning Development, SS&C Technologies, 128 Queen Victoria Street, London, EC4V 4BJ, United Kingdom.

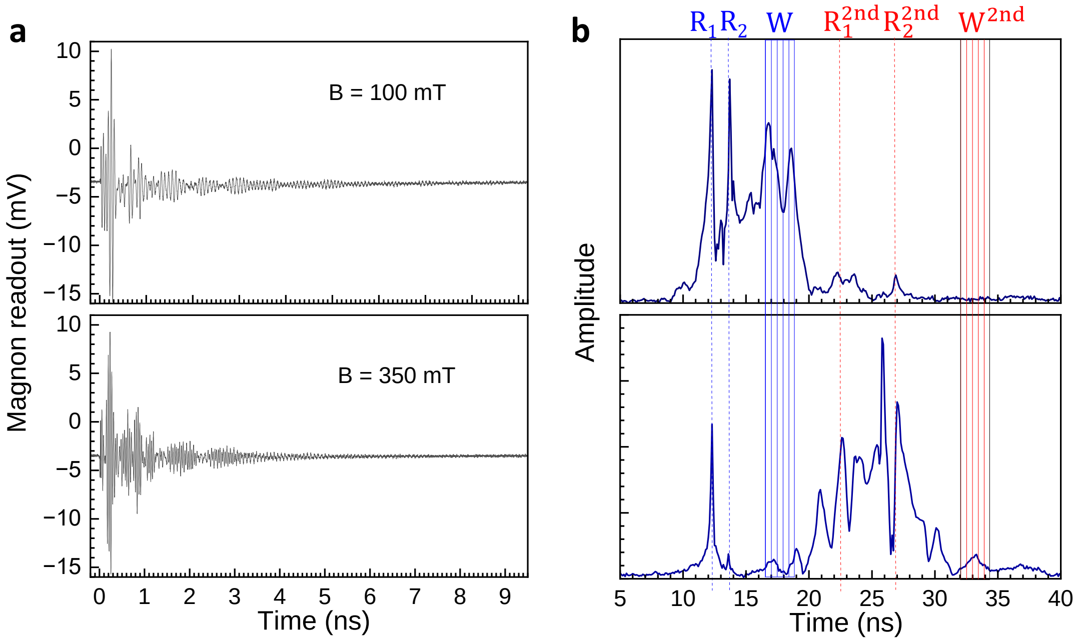

Supplementary Figure 1. **Optically generated phonon and magnon modes.**

Magnon readout signals measured at the spatial overlap of the input and readout laser spots at two strengths of the external magnetic field (**a**) and their fast Fourier transforms (FFT) (**b**). The signals are shown as measured without filtering. The initially generated magnon-phonon wavepacket consists of the first and second order surface phonon modes, R1 (Rayleigh wave) and R2 (Sezawa wave), and the guided modes (W-band) [1], which calculated frequencies are indicated by the blue (first order) and red (second order) dashed lines and patterned rectangular, respectively. The input laser pulse also generates a broad wavepacket of magnon modes [2,3], which form a broad peak in the FFT spectrum. At the 20-µm working distance between the input and readout spots only the contribution of the first-order W-modes remain resolved in the readout signal. However, other modes, which are hidden in the readout noise support the recognition of the visual shapes.

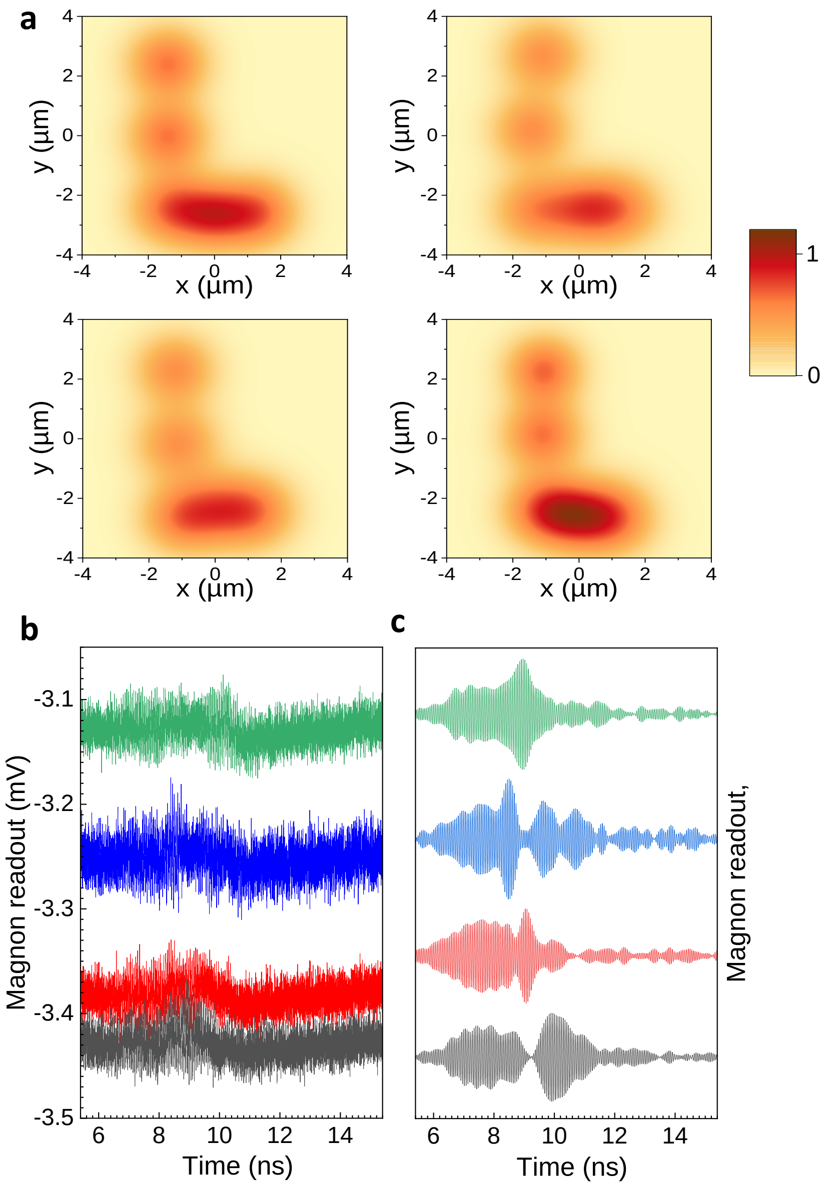

Supplementary Figure 2. **Randomly distorted visual shapes and readout signals.**

a Numerically simulated spatial distribution of the input laser intensity for the symbol ‘L’ obtained by positioning the input laser spots with a randomized error in the range of ±100 nm. **b,c** Four readout signals for the augmented symbol ‘L’: **b** - as measured; **c** - filtered by a 15-20 GHz bandpass filter.

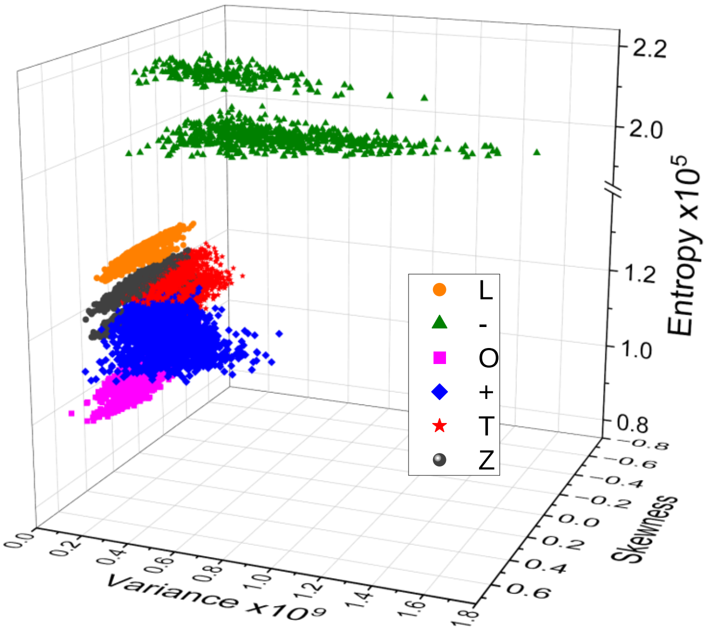

Supplementary Figure 3. Parameters' distribution for the augmented symbols.

Distribution of symbols in the parameter space spanned by the Shannon entropy, the skewness, and the variance, obtained from the waveforms corresponding to the specific relative positions of the 'write' and 'read' laser spots randomly selected from the 10 pre-measured sets (see the main text for details).

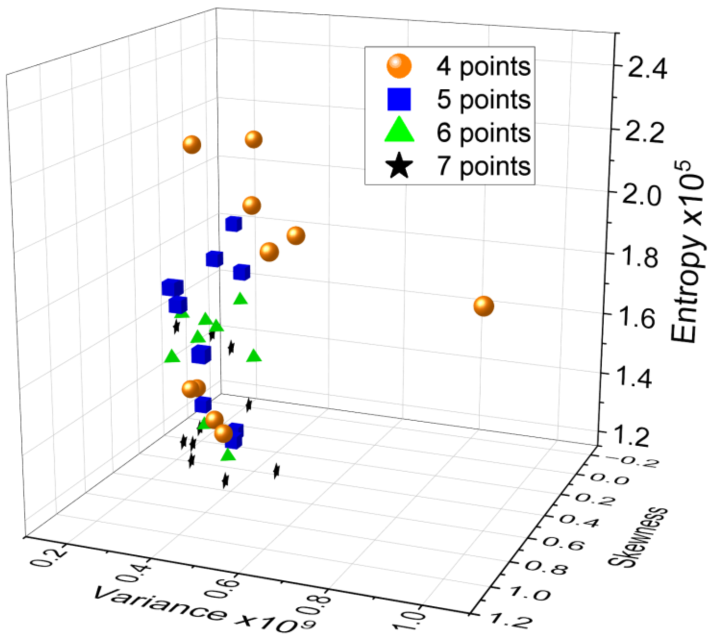

Supplementary Figure 4. Parameters' distribution for the randomized trajectories.

Distribution of the Shannon entropy, the skewness, and the variance for the waveforms corresponding to random trajectories consisting of 4, 5, 6, and 7 discrete relative positions of the 'write' and 'read' laser spots.

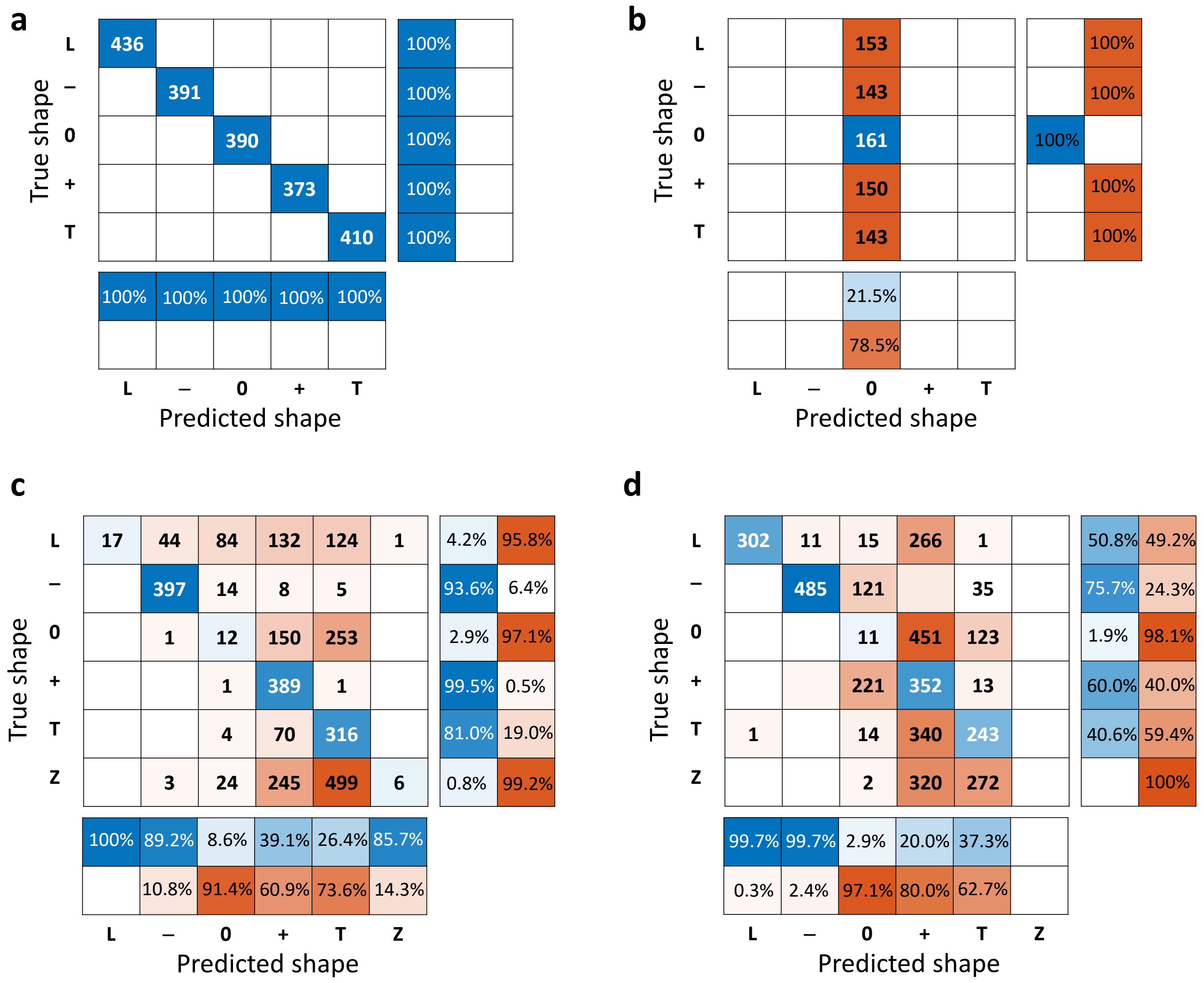

Supplementary Figure 5. **Confusion matrices for the filtered readout signals.**

a. Confusion matrix for unfiltered readout signals when the symbol 'Z' is excluded from consideration. In this case the ANN demonstrates recognition with 100% accuracy. **b.** Confusion matrix for the readout signals passed through the band stop (0- 20 GHz) filter, which makes the recognition impossible. The absence of any values for the symbols in the confusion matrix implies divergence in data processing by ANN **c.** Confusion matrix for the readout signals passed through the band stop (15-20 GHz) filter. In this case the ANN mistakes in 59% cases. Some recognition is still possible due to contribution of the surface (R1 and R2) and low-frequency bulk phonon modes. **d.** Confusion matrix for the readout signals passed through the low pass (<20 GHz) filter. The significant increase of the ANN mistakes up to 43% comparing with the case for the unfiltered signals reflects the contribution of high-order modes and broadband noise to the "visual shapes" recognition.

References

[1] Yaremkevich, D. D. et al., Protected Long-Distance Guiding of Hypersound Underneath a Nanocorrugated Surface. *ACS Nano* **15** 4802-4810 (2021).

[2] Salasysuk A. S. et al., Generation of a localized microwave magnetic field by coherent phonons in a ferromagnetic nanograting, *Phys. Rev. B* **97**, 060404(R) (2018).

[3] Scherbakov A. V. et al., Optical excitation of single- and multi-mode magnetization precession in Galfenol nanolayers”, *Phys. Rev. Appl*. **11**, 031003 (2019).
