# Supplementary material for: On-chip phonon-magnon reservoir for neuromorphic computing
Source: Nat Commun. 2023 Dec 14;14:8296. doi: 10.1038/s41467-023-43891-y (PMC10721880; doi:10.1038/s41467-023-43891-y)
Supplement: Supplementary file 3 — Description of Additional Supplementary Files [file 41467_2023_43891_MOESM3_ESM.docx]

**Description of Additional Supplementary Files**

File Name: SupplementaryVideo1.mov

Description: Calculated (COMSOL Multiphysics) spatial-temporal evolution of the coherent phonon wavepacket after excitation by the write laser pulse. The colour map illustrates the time derivative of the *z*-component of the displacement vector in the ferromagnetic layer.

File Name: Reseroir_SupplementaryInformation.pdf

Description: Supplementary Figure 1. Optically generated phonon and magnon modes.

Supplementary Figure 2. Randomly distorted visual shapes and readout signals.

Supplementary Figure 3. Parameters’ distribution for the augmented symbols.

Supplementary Figure 4. Parameters’ distribution for the randomized trajectories.

Supplementary Figure 5. Confusion matrices for the filtered readout signals.
